# Insegurança alimentar e insegurança hídrica domiciliar: um estudo de base populacional em um município da bacia hidrográfica do Rio Amazonas, Brasil

**DOI:** 10.1590/0102-311XPT125423

**Published:** 2024-05-17

**Authors:** Mayline Menezes da Mata, Adriana Sanudo, Maria Angélica Tavares de Medeiros

**Affiliations:** 1 Instituto Saúde e Sociedade, Universidade Federal de São Paulo, Santos, Brasil.; 2 Universidade Federal do Amazonas, Manaus, Brasil.; 3 Escola Paulista de Medicina, Universidade Federal de São Paulo, São Paulo, Brasil.

**Keywords:** Hunger, Drinking Water, Social Vulnerability, Health Surveys, Amazonian Ecosystem, Fome, Água Potável, Vulnerabilidade Social, Inquéritos Epidemiológicos, Ecossistema Amazônico, Hambre, Agua Potable, Vulnerabilidad Social, Encuestas Epidemiológicas, Ecosistema Amazónico

## Abstract

Este estudo analisa a insegurança alimentar e os fatores a ela associados na área urbana de um município na bacia hidrográfica do Rio Amazonas, Amazônia Ocidental. Trata-se de pesquisa transversal, de base populacional, realizada de agosto a novembro de 2021, com 983 domicílios selecionados por amostragem probabilística estratificada. Empregou-se o modelo de regressão logística multinomial, adotando-se os seguintes critérios: valor de p < 20% na análise bivariada e valor de p < 5% para o ajuste multivariado. Os resultados das análises foram descritos como *odds ratios* (OR) e intervalo de 95% de confiança (IC95%). Foram significantemente associadas à insegurança alimentar leve ou moderada as seguintes variáveis: insegurança hídrica domiciliar, número de moradores ≥ 5 no domicílio, pertencer à classe socioeconômica D ou E, ter pai, mãe ou outro, como chefe da família e ter algum morador beneficiário do Programa Bolsa Família. No modelo de análise para a insegurança alimentar grave constatou-se que viver em insegurança hídrica domiciliar, pertencer à classe socioeconômica D ou E, ter pai, mãe ou outro chefe da família, e tendo este menos que 55 anos, e renda familiar menor que dois salários mínimos aumentaram as chances de insegurança alimentar grave, comparativamente àqueles em segurança alimentar. Em conclusão, verificou-se alta prevalência de insegurança alimentar no Município de Itapiranga, Amazonas, Região Norte do Brasil, associada à situação de vulnerabilidade social e econômica, à falta de serviços públicos e à insegurança hídrica domiciliar.

## Abstract (en)

This study analyzed food insecurity and associated factors in the urban area of a municipality in the Amazon River basin, Western Amazon. This is a cross-sectional population-based study conducted from August to November 2021 with 983 households selected by stratified probability sampling. A multinomial logistic regression model was used, adopting the following criteria: p-value < 20% in the bivariate analysis and p-value < 5% for the multivariate adjustment. The results of the analyses were described as odds ratio (OR) and 95% confidence interval (95%CI). The following variables were significantly associated with mild or moderate food insecurity: household water insecurity; number of residents ≥ 5 in the household; belonging to socioeconomic class D or E; having a father, mother or another as the head of the family; and having any resident as a beneficiary of the Brazilian Income Transfer Program. The analysis model for severe food insecurity showed that living with household water insecurity; belonging to socioeconomic class D or E; having a father, mother or another as the head of the family; age of the head of the family < 55 years; and family income lower that two minimum wages increased the chances of severe food insecurity when compared to those with food security. In conclusion, this study found a high prevalence of food insecurity in the Municipality of Itapiranga, State of Amazonas, North Region of Brazil, associated with social and economic vulnerability, lack of public services, and household water insecurity.

## Resumen

Este estudio analiza la inseguridad alimentaria y los factores asociados, en el área urbana de un municipio de la cuenca hidrográfica del río Amazonas, Amazonia occidental. Se trata de una encuesta transversal, de base poblacional, realizada en el período de agosto a noviembre del 2021, con 983 hogares seleccionados mediante muestreo probabilístico estratificado. Se utilizó el modelo de regresión logística multinomial, adoptando los siguientes criterios: valor de p < 20% en el análisis bivariado y valor de p < 5% para el ajuste multivariado. Los resultados de los análisis se describieron como *odds ratios* (OR) e intervalo de 95% de confianza (IC95%). Las siguientes variables se asociaron significativamente con la inseguridad alimentaria leve o moderada: inseguridad hídrica en el hogar, número de residentes ≥ 5 en el hogar, pertenecer a la clase socioeconómica “D” o “E”, tener padre, madre u otra persona como cabeza de familia y tener a algún residente como beneficiario del Programa Bolsa Familia. En el modelo de análisis para la inseguridad alimentaria severa se encontró que vivir en hogar con inseguridad hídrica, pertenecer a la clase socioeconómica D o E, tener padre, madre u otra persona como cabeza de familia, edad del jefe de familia < 55 años, y un ingreso familiar más bajo que dos salarios mínimos aumentó las probabilidades de sufrir inseguridad alimentaria grave, en comparación con aquellos en situación de seguridad alimentaria. En conclusión, se constató una alta prevalencia de inseguridad alimentaria en el Municipio de Itapiranga, en el interior del Amazonas, Región Norte de Brasil, asociada a la situación de vulnerabilidad social y económica, a la falta de servicios públicos y a la inseguridad hídrica de los hogares.

## Introdução

Em 2022, a insegurança alimentar grave afetou severamente os domicílios da Região Norte do Brasil (25,7%), ratificando disparidades regionais histórico-sociais agravadas pela pandemia da COVID-19 ^1^^,^^2^. Foram devastadores os efeitos da crise sanitária instaurada no país, polo de maior ocorrência de casos e de óbitos por COVID-19 na América Latina e Caribe ^3^. Nesse contexto, sobressai a cidade de Manaus, capital do Estado do Amazonas, como a mais afetada no Norte brasileiro ^4^.

As populações amazônicas vivenciaram, simultaneamente, diferentes formas de insegurança alimentar, referentes à falta de “*acesso regular e permanente a alimentos de qualidade em quantidade suficiente sem comprometer o acesso a outras necessidades essenciais, tendo como base práticas alimentares promotoras de saúde, que respeitem a diversidade cultural e que sejam social, econômica e ambientalmente sustentáveis*” ^5^, além de insegurança hídrica ^2^, expressa pela falta de acesso à água em quantidade segura e suficiente para o consumo humano e o uso domiciliar ^6^.

Tal situação revela o paradoxo da fome e da sede em um bioma que abriga a maior bacia hidrográfica de água doce do planeta, com floresta tropical e vasta biodiversidade ^7^. Na última década, constatam-se impactos promovidos pela crise climática na Região Amazônica, incluindo perda de habitat natural de espécies, redução da biodiversidade e contaminação do solo e das águas. Além disso, o território em questão é marcado por disputas pela terra e violência, em razão da exploração predatória dos recursos naturais e do garimpo ilegal ^8^.

As atipicidades dos fenômenos naturais no contexto amazônico, caracterizadas por eventos extremos, como secas e enchentes ^9^, além de causarem perdas econômicas significativas, impactam na segurança alimentar e na segurança hídrica. Isso porque ambos os agravos se associam sinergicamente a: baixa renda, transtornos mentais, dificuldade financeira e fatores demográficos ^10^^,^^11^, ampliando os riscos à sobrevivência dessa população. Destarte, essa dura realidade ameaça a meta mundial de eliminar a fome e todas as formas de má nutrição até 2030, no âmbito dos Objetivos de Desenvolvimento Sustentável (ODS).

Compreender as razões para a exclusão das populações amazônicas de inquéritos epidemiológicos nacionais, como distribuição espacial, baixa densidade demográfica e dificuldade de acesso ^12^, é tarefa imprescindível para enfrentar os desafios que invisibilizam esses povos. Para tanto, entende-se que a produção de evidências científicas assume papel estratégico para a condução de políticas públicas regionalizadas, direcionadas ao meio ambiente e às pessoas que ocupam esse território ^13^.

Neste estudo tem-se o objetivo de analisar a insegurança alimentar em associação à insegurança hídrica, aos fatores sociodemográficos e ao acesso a programas sociais de transferência de renda em domicílios da área urbana do Município de Itapiranga (Amazonas), situado na bacia hidrográfica do Rio Amazonas, na Amazônia Ocidental.

## Material e métodos

### Delineamento e local do estudo

Trata-se de estudo transversal, de base populacional, realizado em domicílios da área urbana no Município de Itapiranga, no período de agosto a novembro de 2021.

O Município de Itapiranga localiza-se na bacia hidrográfica do Rio Amazonas, com população estimada, em 2021, de aproximadamente 9.312 habitantes, com densidade demográfica de 1,94 habitantes/km^2^ e Índice de Desenvolvimento Humano (IDH) médio de 0,654, distante 226km de Manaus, com acesso fluvial (12 horas de viagem) e terrestre (6 horas de viagem) ^14^. O transporte fluvial no município é utilizado particularmente para o abastecimento do comércio local.

### Plano amostral

A população do estudo foi selecionada por amostragem probabilística estratificada. Para identificar a população de referência utilizou-se o cadastro do Sistema de Informação da Atenção Básica (SIAB), da Secretaria Municipal da Saúde (SEMSA) de Itapiranga de 2021, maior fonte de dados disponível, correspondente às três áreas de saúde, entre as quais se organiza o Sistema Único de Saúde (SUS) local, a saber: área 04 (1.210 habitantes), área 05 (2.255 habitantes) e área 06 (1.682 habitantes). A população de interesse concentrou-se na faixa etária entre 20 e 59 anos, considerando uma prevalência de 38,4% de domicílios em insegurança hídrica na Região Norte ^15^. Por se tratar de dois desfechos principais para o inquérito realizado (insegurança alimentar e insegurança hídrica), escolheu-se o que apresentou maior tamanho amostral, a saber, o de insegurança hídrica. Estabeleceu-se um erro absoluto de 5%, com 95% de confiança, para estimar a prevalência de insegurança hídrica. Além disso, foram acrescidos 10% de indivíduos no cálculo final da amostra, para minimizar possíveis perdas e/ou recusas para cada uma das três áreas de saúde do município, respectivamente 308, 345 e 330 indivíduos, o que resultou em um total de 983 pessoas.

Consideraram-se elegíveis todos os domicílios com a presença de, pelo menos, um morador adulto com idade entre 18 e 59 anos, exceto mulheres grávidas e pessoas com deficiência (PCD).

### Coleta de dados

A coleta de dados foi realizada por entrevistadores capacitados, sob supervisão direta da auxiliar de pesquisa. As entrevistas ocorreram presencialmente, respeitando-se os protocolos sanitários de biossegurança, com um adulto respondente selecionado por cada domicílio. Para aqueles domicílios com mais de um adulto elegível presente no ato da entrevista, realizou-se sorteio aleatório. Para garantir a qualidade dos dados coletados, realizou-se supervisão contínua e sistemática do trabalho de campo. Os questionários foram revisados e, quando necessário, o retorno ao campo foi imediato. Isso assegurou um percentual de resposta de 100%.

### Variáveis do estudo

A variável de desfecho deste estudo foi a (in)segurança alimentar, medida com o auxílio da *Escala Brasileira de Insegurança Alimentar* (EBIA), em sua versão curta de oito itens e validada ^16^^,^^17^. A partir da soma desses oito itens classificou-se os respondentes em três categorias: segurança alimentar (EBIA = 0), insegurança alimentar leve/moderada (EBIA entre 1 e 5 pontos), e insegurança alimentar grave (EBIA entre 6 e 8 pontos).

Como variáveis independentes foram consideradas:

Número de moradores no domicílio (0-4 e 5 ou mais);

Presença de crianças (não ou sim);

Variáveis demográficas: sexo, idade em anos (< 55 e ≥ 55), raça/cor da pele autodeclarada, chefe do domicílio (pessoa de referência no domicílio: pai, mãe, outro ou avós);

Variáveis socioeconômicas: renda familiar, avaliada em salários mínimos (até 2 e > 2), e classe socioeconômica ^18^. Tal classificação baseia-se em: nível educacional do chefe da família, número de bens de consumo (como televisão, banheiros, automóveis, microcomputador, lava louça, geladeira, *freezer*, lava roupa, DVD *players*, micro-ondas, motocicleta e secadora de roupa); número de trabalhadores domésticos, grau de instrução do chefe da família e acesso a serviços públicos (água encanada e rua pavimentada). Isso posto, classificam-se os indivíduos em subgrupos de A-E (sendo A a classe mais alta). Para facilitar a interpretação e melhorar a acurácia das estimativas do modelo de regressão, categorias com baixas frequências foram agrupadas. Assim, as classes socioeconômicas A, B, e C1 foram reunidas, o mesmo ocorrendo com as classes C2, D e E.

Acesso a política pública (Programa Bolsa Família);

(In)segurança hídrica domiciliar, medida pela *Household Water Insecurity Experiences Scale* (HWISE; Escala de Insegurança Hídrica Domiciliar), validada internacionalmente. O desenvolvimento e a validação da HWISE envolveram amostras de 29 locais, em 23 países de baixa e média renda, incluindo o Brasil ^19^. Ressalta-se que a versão aplicada neste estudo foi a traduzida pelos autores responsáveis pelo desenvolvimento e pela validação da escala, que é composta por 12 questões correspondentes às últimas quatro semanas, considerando pontos de corte estabelecidos com base nos escores de respostas aos itens avaliados em: segurança hídrica (0-11 pontos) e insegurança hídrica (12-36 pontos). Para a classificação final dos domicílios adotou-se a estratificação dos níveis de segurança/insegurança hídrica, correspondente à soma da pontuação dos 12 quesitos, variando em uma amplitude de 0 a 36. Os itens não respondidos invalidaram a medida da insegurança hídrica para aquele domicílio.

### Análise dos dados

**Figura 1:**
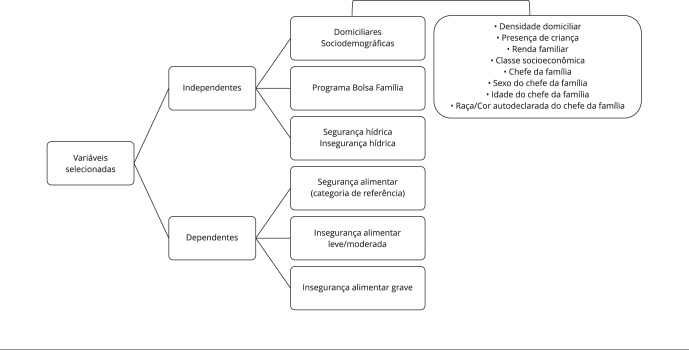
Variáveis empregadas nos modelos brutos e ajustados. Itapiranga, Amazonas, Brasil, 2023.

Inicialmente realizou-se análise descritiva simples, com frequências absolutas e relativas, e estratificada por nível de segurança e insegurança alimentar segundo as respectivas variáveis: densidade domiciliar, presença de criança no domicílio, classe socioeconômica, chefe do domicílio, sexo, idade, raça/cor da pele autodeclarada, renda familiar, classe socioeconômica, Programa Bolsa Família e (in)segurança hídrica domiciliar. Para verificar a existência de associações à variável resposta utilizou-se o teste qui-quadrado (Figura 1).

Nota: na seleção das variáveis considerou-se a plausibilidade teórica entre as variáveis explicativas (sociodemográficas e domiciliares, participação em programas governamentais e a (in)segurança hídrica, medida pela *Household Water Insecurity Experiences Scale* - HWISE; Escala de Insegurança Hídrica Domiciliar) e as variáveis respostas (insegurança alimentar leve e moderada e insegurança alimentar grave), tendo a segurança alimentar como unidade de referência. Realizou-se análise descritiva e investigou-se a existência de associação mediante o teste qui-quadrado, incialmente. Para analisar os fatores associados em ambos os desfechos, estimou-se a medida de efeito por *odds ratios* (OR), mediante regressão logística multinomial, adotando-se como critérios valor de p < 20% na análise bruta e valor de p < 5% para a ajustada, e intervalos de 95% de confiança (IC95%).

Para identificar os fatores associados à insegurança alimentar, estimou-se a medida de efeito por *odds ratios* (OR), mediante regressão logística multinomial. As associações entre insegurança alimentar (leve/moderada e grave) e variáveis independentes foram verificadas pelo cálculo de estimativas brutas e ajustadas, com intervalos de 95% de confiança (IC95%) e assumindo a segurança alimentar como categoria de referência (Figura 1).

A seleção dos potenciais variáveis foi feita pelo respectivo critério estatístico, a saber: variáveis que apresentaram valor de p < 20% na análise bivariada foram selecionadas para o ajuste multivariado. No modelo ajustado, variáveis que apresentaram valor de p > 5% foram excluídas uma a uma, até alcançar o modelo final ajustado, mantendo-se apenas variáveis que apresentaram significância estatística.

As análises estatísticas foram realizadas no programa Stata/SE 17 (https://www.stata.com) e todos os testes foram bilaterais.

### Aspectos éticos

O estudo foi aprovado pelo Comitê de Ética da Universidade Federal de São Paulo (CAAE 55669522.5.0000.5505). Os participantes que consentiram com a participação assinaram o Termo de Consentimento Livre e Esclarecido (TCLE), consoante à *Declaração de Helsinque*, conforme preconiza a *Resolução nº 466/2012* do Conselho Nacional de Saúde.

## Resultados

Dos 983 domicílios incluídos, em aproximadamente 70% havia até quatro moradores, sem crianças (54,9%); as classes socioeconômicas mais frequentes foram D ou E (61,3%). Os chefes apresentaram, majoritariamente, menos que 55 anos (73,8%), autodeclararam-se pardos ou pretos (89,5%), com renda familiar de até dois salários mínimos (90,4%). Em mais da metade desses domicílios (55,5%) algum morador recebia o auxílio do Programa Bolsa Família; 30,7% foram classificados em segurança alimentar, 12,8% em insegurança alimentar leve e 19,8% em insegurança alimentar moderada. A insegurança alimentar grave afetou 36,7% (IC95%: 29,7; 35,6) e a insegurança hídrica 46,1% (IC95%: 43,0; 49,4), conforme a Tabela 1.

**Tabela 1:** Distribuição dos dados sociodemográficos dos residentes em área urbana e da unidade domiciliar, (in)segurança hídrica domiciliar, segundo os níveis de (in)segurança alimentar. Itapiranga, Amazonas, Brasil, 2023.

Variáveis	Total (N = 982)		EBIA						Valor de p *
			Segurança alimentar (n = 302; 30,8%)		Insegurança alimentar leve/moderada (n = 320; 32,6%)		Insegurança alimentar grave (n = 360; 36,7%)		
	n	%	n	%	n	%	n	%	
Número de moradores no domicílio									0,001
0-4	690	70,3	237	78,5	216	67,5	237	65,8	
5 ou mais	292	29,7	65	21,5	104	32,5	123	34,2	
Criança no domicílio									0,002
Não	539	54,9	187	61,9	178	55,6	174	48,3	
Sim	153	15,6	115	38,1	142	44,4	186	51,7	
Classe socioeconômica									< 0,001
A ou B ou C1	119	12,1	61	20,2	40	12,5	18	5,0	
C2	261	26,6	105	34,8	88	27,5	68	18,9	
D ou E	602	61,3	136	45,0	192	60,0	274	76,1	
Chefe da família									< 0,001
Pai ou mãe ou outro	943	96,0	276	91,4	312	97,5	355	98,6	
Avós	39	4,0	26	8,6	8	2,5	5	1,4	
Sexo do chefe da família									0,655
Masculino	656	66,8	200	66,2	220	68,8	236	65,6	
Feminino	326	33,2	102	33,8	100	31,2	124	34,4	
Idade do chefe da família (anos)									< 0,001
< 55	725	73,8	198	65,6	231	72,2	296	82,2	
≥ 55	257	26,2	104	34,4	89	27,8	64	17,8	
Raça/Cor da pele do chefe da família									0,196
Parda ou preta	879	89,5	263	87,1	287	89,7	329	91,4	
Branca ou amarela	103	10,5	39	12,9	33	10,3	31	8,6	
Renda total da família (salários mínimos)									< 0,001
Até 2	888	90,4	262	86,8	278	86,9	348	96,7	
> 2	94	9,6	40	13,2	42	13,1	12	3,3	
Programa Bolsa Família									< 0,001
Sim	544	55,5	152	50,3	158	49,5	234	65,0	
Não	437	44,5	150	49,7	161	50,5	126	35,0	
HWISE									< 0,001
Segurança hídrica	529	53,9	225	74,5	174	54,4	130	36,1	
Insegurança hídrica	453	46,1	77	25,5	146	45,6	230	63,9	

EBIA: *Escala Brasileira de Insegurança Alimentar*; HWISE: *Household Water Insecurity Experiences Scale* (Escala de Insegurança Hídrica Domiciliar).

* Teste qui-quadrado.

À exceção do sexo e da cor do chefe da família, as demais variáveis se associaram estatisticamente aos níveis de segurança e insegurança alimentar. Nos domicílios cujos residentes se encontravam em insegurança alimentar grave, aproximadamente, 52% contavam com crianças, 76% pertenciam às classes socioeconômicas D ou E, e aproximadamente, 64% estavam em situação de insegurança hídrica, segundo o teste qui-quadrado (Tabela 1).

De acordo com a Tabela 2, ter cinco ou mais moradores no domicílio, pertencer às classes D ou E, ter pai, mãe ou outro indivíduo como chefe do domicílio e estar em insegurança hídrica aumentou as chances de insegurança alimentar (p < 0,05).

**Tabela 2:** Análise bivariada da associação entre níveis de segurança e insegurança alimentar *, segundo a *Escala Brasileira de Insegurança Alimentar* (EBIA), variáveis sociodemográficas do domicílio e (in)segurança hídrica. Itapiranga, Amazonas, Brasil, 2023.

Variáveis	Análise bruta			
	**Insegurança alimentar leve/moderada *vs.* segurança alimentar**		**Insegurança alimentar grave *vs.* segurança alimentar**	
	OR_bruto_	IC95%	OR_bruto_	IC95%
Número de moradores no domicílio: 5 ou mais x 0-4	1,756	1,222; 2,518	1,892	1,333; 2,687
Criança no domicílio: sim x não	1,297	0,942; 1,787	1,738	1,273; 2,373
Classe socioeconômica:				
C2 x A ou B ou C1	1,278	0,783; 2,085	2,195	1,195; 4,032
D ou E x A ou B ou C1	2,153	1,365; 3,395	6,828	3,881; 12,010
Chefe da família: pai ou mãe ou outros x avós	3,674	1,636; 8,252	6,688	2,534; 17,651
Sexo do chefe da família: feminino x masculino	0,891	0,637; 1,247	1,030	0,746; 1,423
Idade do chefe da família (anos): < 55 x ≥ 55	1,363	0,969; 1,917	2,429	1,695; 3,481
Raça/Cor da pele do chefe da família: parda ou preta x branca ou amarela	1,290	0,788; 2,112	1,574	0,956; 2,592
Renda total da família (salários mínimos): até 2 x > 2	1,011	0,635; 1,609	4,427	2,277; 8,610
Programa bolsa família: sim x não	0,968	0,707; 1,327	1,833	1,340; 2,506
HWISE: insegurança x segurança hídrica	2,452	1,745; 3,441	5,170	3,692; 7,239

HWISE: *Household Water Insecurity Experiences Scale* (Escala de Insegurança Hídrica Domiciliar); IC95%: intervalo de 95% de confiança; OR: *odds ratio*.

* Segurança alimentar como categoria de referência.

Após o ajuste multivariado, verificou-se que nos domicílios com cinco ou mais moradores pertencentes às classes D ou E, com pai, mãe ou outro como chefe da família, titular do Programa Bolsa Família e em insegurança hídrica houve maiores chances de insegurança alimentar leve ou moderada (Tabela 3). Domicílios com cinco ou mais moradores, pertencentes às classes D ou E, com pai, mãe ou outro como chefe da família, com idade do chefe menor que 55 anos, renda familiar menor que dois salários mínimos e em insegurança hídrica apresentaram maiores chances de estar em insegurança alimentar grave (Tabela 4).

**Tabela 3:** Análise multivariada da associação entre níveis de insegurança alimentar leve/moderada *vs.* segurança alimentar, segundo a *Escala Brasileira de Insegurança Alimentar* (EBIA), variáveis sociodemográficas do domicílio e (in)segurança hídrica. Itapiranga, Amazonas, Brasil, 2023.

Variáveis	**Insegurança alimentar leve/moderada *vs.* Segurança alimentar**			
	Modelo inicial		Modelo final	
	OR_bruto_	IC95%	OR_bruto_	IC95%
Número de moradores no domicílio: 5 ou mais x 0-4	1,755	1,165; 2,642	1,758	1,192; 2,593
Criança no domicílio: sim x não	1,005	0,693; 1,458		
Classe socioeconômica:				
C2 x A ou B ou C1	1,336	0,805; 2,217	1,334	0,804; 2,213
D ou E x A ou B ou C1	2,442	1,481; 4,024	2,440	1,483; 4,014
Chefe da família: pai ou mãe ou outros x avós	3,443	1,387; 8,547	3,426	1,395; 8,415
Idade do chefe da família (anos): < 55 x ≥ 55	1,174	0,795; 1,735	1,174	0,798; 1,727
Raça/cor da pele do chefe da família: parda ou preta x branca ou amarela	1,021	0,596; 1,749		
Renda total da família (salários mínimos): até 2 x > 2	0,796	0,470; 1,349	0,793	0,471; 1,337
Programa Bolsa Família: sim x não	1,443	1,004; 2,075	1,442	1,010; 2,059
HWISE: insegurança x segurança hídrica	2,181	1,539; 3,091	2,182	1,541; 3,089

HWISE: *Household Water Insecurity Experiences Scale* (Escala de Insegurança Hídrica Domiciliar); IC95%: intervalo de 95% de confiança; OR: *odds ratio*.

**Tabela 4:** Análise multivariada da associação entre níveis de insegurança alimentar grave *vs.* segurança alimentar, segundo a *Escala Brasileira de Insegurança Alimentar* (EBIA), variáveis sociodemográficas do domicílio e (in)segurança hídrica. Itapiranga, Amazonas, Brasil, 2023.

Variáveis	**Insegurança alimentar grave *vs.* Segurança alimentar**			
	Modelo inicial		Modelo final	
	OR_bruto_	IC95%	OR_bruto_	IC95%
Número de moradores no domicílio: 5 ou mais x 0-4	1,505	0,988; 2,293	1,531	1,031; 2,273
Criança no domicílio: sim x não	1,069	0,728; 1,571		
Classe socioeconômica:				
C2 x A ou B ou C1	1,752	0,913; 3,362	1,741	0,910; 3,331
D ou E x A ou B ou C1	5,223	2,794; 9,766	5,186	2,787; 9,651
Chefe da família: pai ou mãe ou outros x avós	3,903	1,304; 11,681	3,781	1,273; 11,223
Idade do chefe da família (anos): < 55 x ≥ 55	1,827	1,205; 2,771	1,840	1,218; 2,780
Raça/Cor da pele do chefe da família: parda ou preta x branca ou amarela	1,138	0,618; 2,094		
Renda total da família (salários mínimos): até 2 x > 2	2,606	1,242; 5,465	2,562	1,222; 5,373
Programa Bolsa Família: sim x não	0,961	0,657; 1,404	0,949	0,654; 1,378
HWISE: insegurança x segurança hídrica	4,349	3,054; 6,194	4,361	3,062; 6,210

HWISE: *Household Water Insecurity Experiences Scale* (Escala de Insegurança Hídrica Domiciliar); IC95%: intervalo de 95% de confiança; OR: *odds ratio*.

## Discussão

Neste inquérito de base populacional, realizado durante a pandemia de COVID-19, analisou-se a associação entre insegurança alimentar e os fatores insegurança hídrica domiciliar, densidade domiciliar e características sociodemográficas do chefe do domicílio na área urbana no Município de Itapiranga. A alta prevalência de insegurança alimentar e a inter-relação entre esse agravo e a insegurança hídrica vão além do olhar estrito de saúde pública, já que insegurança alimentar e insegurança hídrica são condições complexas e multifatoriais, que abrangem o modelo excludente de desenvolvimento econômico e social brasileiro.

A insegurança alimentar grave acometeu uma proporção considerável da população, frisando-se que naqueles domicílios com pessoas em situação de insegurança alimentar aumentaram as chances de ocorrência de insegurança hídrica. No âmbito nacional, a insegurança alimentar grave intensificou-se expressivamente entre 2013 e 2018 ^20^, crescimento correspondente a 269%, com declínio da proporção de domicílios em segurança alimentar (46,4%) ^21^. Esse aumento decorreu de um conjunto de políticas governamentais pautadas em medidas de austeridade fiscal implantadas desde 2015, com forte incidência sobre o desmonte das políticas públicas de garantia de direitos, como o Direito Humano à Alimentação Adequada (DHAA) e à segurança alimentar e nutricional, e o desmantelamento do Sistema Nacional de Segurança Alimentar e Nutricional (SISAN) ^22^^,^^23^. Tal cenário foi agravado pelas crises econômica, política e sanitária, com incremento do desemprego e ampliação das desigualdades sociais, determinados pela negligência governamental no enfrentamento da pandemia de COVID-19, o que conduziu o país a uma tragédia sanitária sem precedentes ^1^^,^^2^.

A alta prevalência de insegurança alimentar diagnosticada neste estudo, sobretudo na forma grave, foi reportada em outro inquérito de base populacional, realizado em 2018, no período pré-pandêmico, em um município do Amazonas, na região do Médio Solimões, quando se encontrou uma prevalência de insegurança alimentar de 76,5% ^24^. Ademais, os achados desta pesquisa corroboram as proporções de insegurança alimentar e insegurança alimentar grave em áreas rurais brasileiras (2008-2017), que variaram de 32,2% a 88,8%, e de 3% a 39,5%, respectivamente ^25^.

Outrossim, os fatores associados à insegurança alimentar, já descritos na literatura científica ^26^^,^^27^^,^^28^^,^^29^, refletindo um contexto de vulnerabilidade e invisibilidade da população do Amazonas, e somados à preexistente concentração dos sistemas de abastecimento, transporte, provisão de serviços e à condução da vida política em Manaus ^30^, impactaram negativamente as políticas de segurança alimentar e nutricional, necessárias para dirimir desigualdades sociais e contribuir com a promoção de condições de vida dignas ^23^.

Percebe-se, portanto, que a situação das áreas urbanas dos municípios do Amazonas, a exemplo de Itapiranga, assemelha-se às das áreas rurais brasileiras, no que se refere às vulnerabilidades socioeconômicas, alimentar e nutricional. Contudo, destaca-se que no Amazonas a associação direta entre área rural e produção agrícola se rompe, uma vez que, na zona rural, tal produção assume características próprias, particularidades manifestas no tempo e nos caminhos das águas, centradas na conservação da biodiversidade e na territorialidade dos povos ^31^.

A insegurança alimentar e a insegurança hídrica são problemáticas urgentes em países de baixa e média renda do Sul Global, fenômeno que afeta sobremaneira as populações mais vulneráveis ^32^. No cenário pandêmico brasileiro, estima-se que, em 2022, 33,1 milhões de pessoas passaram fome ^2^. Na Região Norte, a insegurança alimentar moderada/grave foi cinco vezes maior, expressando intensas disparidades regionais quando comparada às demais regiões do país, com melhores indicadores socioeconômicos ^1^.

A associação entre insegurança alimentar e insegurança hídrica, verificada nesta pesquisa, traz à tona a precariedade das condições de saneamento básico e de serviços públicos em geral nos municípios do interior do Amazonas, contrastando com a capital, Manaus, que detém o maior contingente populacional (aproximadamente 2.255.903 pessoas), um IDH médio de 0,737, e concentra oferta de serviços. Apesar disso, apenas 62,4% da cidade é coberta por serviço de tratamento de esgoto adequado. Já no Município de Itapiranga, somente 6,5% dos domicílios dispõem de rede de saneamento básico ^14^.

Em estudo em que se analisou a cobertura do abastecimento e a qualidade da água distribuída no Brasil, em 2019, verificou-se que somente 58% da Região Norte foi atendida por serviços de água, e parte da população está exposta aos riscos relacionados à qualidade da água ^33^. Já no Amazonas, apenas a microrregião de Manaus reuniu os melhores parâmetros químicos, físicos e microbiológicos, em relação ao conjunto de indicadores da microrregião do interior, revelando fragilidades na gestão da vigilância da qualidade da água disponível para o consumo humano no estado ^34^.

O fornecimento adequado de água, em termos de quantidade e qualidade, é crucial para o desenvolvimento socioeconômico, com reflexos diretos sobre as condições de saúde, a prevenção de agravos e o bem-estar da população ^33^. Desse modo, considerar o papel das questões hídricas sobre a insegurança alimentar pode favorecer a constituição e a implementação das políticas públicas mais efetivas ^6^.

Neste estudo, observou-se que os domicílios submetidos à insegurança alimentar, especialmente nas formas moderada e grave, tiveram maiores chances de apresentar insegurança hídrica. Esse dado se assemelha ao apontado por Young et al. ^6^, que, ao investigarem a insegurança hídrica e a insegurança alimentar em 25 países de baixa e média renda, concluíram que a probabilidade de o indivíduo experimentar a insegurança alimentar moderada e grave é maior entre os que também experimentaram a insegurança hídrica.

Isso posto, a insegurança hídrica domiciliar aqui revelada configura grave problema de saúde pública no Município de Itapiranga, visto que a água, além de direito humano básico, é um nutriente essencial à vida ^32^. Tal achado indica uma questão até então não reportada no interior da Amazônia brasileira. Embora tenham sido realizadas investigações acerca da qualidade, distribuição e da vigilância da água para o consumo humano na região ^7^^,^^8^^,^^33^^,^^34^, inexistem registros na literatura científica que analisaram a experiência da insegurança hídrica domiciliar.

Na escala de insegurança hídrica domiciliar (HWISE), consideram-se a experiência associada às dificuldades de acesso à água no domicílio nas últimas quatro semanas, a disponibilidade (interrupção do abastecimento), o uso da água para lavagem de roupa, mudança de rotina, consumo de água e uso para cocção de alimentos, higienização de mãos e banho, assim como perguntas referentes a questões emocionais decorrentes da insegurança hídrica, como preocupação, raiva ou vergonha ^19^. Assim, é possível refletir acerca do nexo água-nutriente-segurança alimentar para a saúde ^35^.

A prevalência de insegurança hídrica no Município de Itapiranga foi superior à encontrada na região da África subsaariana que, em 2020, correspondeu a 36,1%, associadamente à baixa renda familiar, residir em regiões periféricas de centros urbanos e sob os efeitos graves da pandemia da COVID-19 ^11^. Brewis et al. ^36^, ao analisarem os efeitos da insegurança hídrica em 21 países, sugeriram que a insegurança hídrica domiciliar coexiste cronicamente com a insegurança alimentar domiciliar.

Outro ponto que merece destaque é a vulnerabilidade social existente no município de estudo, convergente com o fenômeno da transição demográfica no Brasil, que é marcada por disparidades regionais ^37^. A desigualdade econômica, neste estudo, é revelada pela alta proporção de famílias pertencentes às classes socioeconômicas D e E, com baixa renda familiar (em média R$ 954,00 em 2021) e expressiva participação em programas de transferência condicionada de renda. Com isso, reivindica-se o papel estratégico do Estado, para incidir sobre os efeitos deletérios das históricas discrepâncias inter-regionais brasileiras, que são anteriores ao período pandêmico, e que foram agudizadas pela desproteção social consequente à pandemia ^38^^,^^39^.

O Programa Bolsa Família, um dos mais vultosos programas de transferência condicionada de renda, mundialmente falando ^40^, abarca importante número de participantes, constituindo-se em política fundamental para o enfrentamento das iniquidades no município. Os efeitos do programa ressoam positivamente sobre a redução da mortalidade infantil e o maior acesso aos serviços de atenção primária à saúde (APS), à alimentação, à maior frequência escolar e à redução da evasão escolar, ainda que não tenham sido observadas a interrupção do ciclo intergeracional da pobreza e a melhoria do estado nutricional das famílias beneficiárias ^40^. Registra-se que esses resultados positivos da participação no programa e a melhoria das condições socioeconômicas das populações envolvidas concorrem para a redução da ocorrência da insegurança alimentar, especialmente em sua forma grave, a fome ^41^. Daí o reconhecimento do Bolsa Família como programa estruturante do SISAN, visando gerar autonomia e garantir o acesso à alimentação via transferência de renda.

### Limitações e avanços da pesquisa

Este estudo apresenta limitações que precisam ser discutidas. Em primeiro plano, relacionam-se ao tipo de investigação, de natureza transversal, que impossibilita inferir causalidade. Em segundo plano, para os dados utilizados no cálculo amostral, considerou-se a população coberta pelo SUS em 2021. Em compensação, tais informações, de âmbito local, configuram-se como as mais abrangentes e fidedignas, visto que o recenseamento do Instituto Brasileiro de Geografia e Estatística (IBGE) iniciou-se somente em 2022, com dois anos de atraso, em razão dos cortes orçamentários federais, paralelamente à crise sanitária. Ressalta-se também que, nos últimos 12 anos, o Município de Itapiranga sofreu diversas transformações demográficas e de ocupação territorial. Nesse período, foram criados três novos bairros com considerável contingente populacional, e a expansão da cobertura da APS correspondeu a 100% da população em 2020 ^42^.

Em contrapartida, a principal inovação da pesquisa consiste no fato de ser o primeiro inquérito a abordar a insegurança alimentar, que atinge severamente a Região Norte do país, verificando a existência de associações entre o fenômeno da insegurança alimentar grave e a insegurança hídrica domiciliar a partir de escalas de experiência domiciliar usadas para medir o acesso aos alimentos e à água em área urbana de um município da Amazônia brasileira.

## Conclusão

Os resultados deste estudo corroboram a existência de elevada prevalência de insegurança alimentar no interior do Amazonas, Região Norte do Brasil, associadamente às iniquidades sociais, à escassez de serviços públicos e à insegurança hídrica domiciliar. Tal circunstância pode ter sido acentuada pela pandemia da COVID-19, que no Amazonas assumiu as piores proporções em termos de morbimortalidade relativamente aos demais estados brasileiros, mas é anterior a ela.

Os achados inéditos aqui apresentados oportunizam investimentos no campo da agenda pública de segurança alimentar e nutricional, uma vez que o diagnóstico da insegurança alimentar e da insegurança hídrica no Município de Itapiranga poderá contribuir com a proposição de ações efetivas nessa direção. Ademais, espera-se que as evidências apresentadas mobilizem a gestão pública para qualificar e fortalecer a Política Nacional de Segurança Alimentar e Nutricional (PNSAN) no âmbito municipal, envolvendo a construção de políticas intersetoriais, imprescindíveis para o enfrentamento desse quadro. Finalmente, sugere-se o delineamento de novas pesquisas, com instrumentos validados para a realidade amazônica, vislumbrando-se a superação dos desafios inerentes a esse território.

## References

[1] Bagni UV, Rodrigues AA, Ribeiro ECSA, Salles-Costa R, Ferreira AA (2022). Food insecurity in households with persons with disabilities in a
situation of extreme vulnerability in Brazil a secondary cross-sectional
analysis. Lancet Reg Health Am.

[2] Rede Brasileira de Pesquisa em Soberania e Seguranc¸a Alimentar e
Nutricional II VIGISAN National Survey on Food Insecurity in the Context of the
Covid-19 Pandemic in Brazil..

[3] Word Health Organization WHO COVID-19 dashboard.. https://covid19.who.int.

[4] Orellana JDY, Cunha GM, Marrero L, Moreira RI, Leite IC, Horta BL (2021). Excesso de mortes durante a pandemia de COVID-19 subnotificação e
desigualdades regionais no Brasil. Cad Saúde Pública.

[5] Conselho Nacional de Segurança Alimentar e Nutricional II Conferência Nacional de Segurança Alimentar e Nutricional, 17-20
março, 2004..

[6] Young SL, Bethancourt HJ, Frongillo EA, Vivianic S, Cafieroc C (2023). Concurrence of water and food insecurities, 25 low- and
middle-income countries. Bull World Health Organ.

[7] Cardozo M, Diniz MB, Szlafsztein CF (2022). Amazon Basin water resources ecosystem services on the approach
of global public goods. Agua y Territorio.

[8] Escada MIS, Amaral S, Fernandes DA (2023). Dinâmicas de ocupação e as transformações das paisagens na
Amazônia, Brasil. Cad Saúde Pública.

[9] Costa MSB, Fraxe TJP, Norte AF, Oka JM, Carneiro JPR, Gonçalves VVC (2023). Percepção da comunidade local sobre os efeitos da mortandade de
peixes no lago do Rei no Careiro da Várzea-Amazonas. Res Soc Dev.

[10] Brewis A, Choudharyb N, Wuticha A (2019). Household water insecurity may influence common mental disorders
directly and indirectly through multiple pathways evidence from
Haiti. Soc Sci Med.

[11] Young SL, Bethancourt HJ, Ritter ZR, Frongillo EA (2022). Estimating national, demographic, and socioeconomic disparities
in water insecurity experiences in low- and middle-income countries in
2020-2021: a cross-sectional, observational study, using nationally
representative data.. Lancet Planet Health.

[12] Rezende AAB, Silva RP, Pedrosa NL, Luz RA, Paixão AN, Rodrigues W (2023). Distribuição da COVID-19 e dos recursos de saúde na Amazônia
Legal uma análise espacial. Ciênc Saúde Colet.

[13] D'Antona AO (2023). Environmental conservation, spatial mobility, and living
conditions of traditional populations in protected areas: for adequate
health access models in the Amazonian context.. Cad Saúde Pública.

[14] Instituto Brasileiro de Geografia e Estatística Amazonas..

[15] Rede Brasileira de Pesquisa em Soberania e Segurança Alimentar e
Nutricional I VIGISAN National Survey on Food Insecurity in the Context of the
Covid-19 Pandemic in Brazil..

[16] Interlenghi GS, Reichenheim ME, Segall-Corrêa AM, Pérez-Escamilla R, Moraes CL, Salles-Costa R (2019). Suitability of the eight-item version of the Brazilian Household
Food Insecurity Measurement Scale to identify risk groups: evidence from a
nationwide representative sample.. Public Health Nutr.

[17] Segall-Corrêa AM, Marin-León L, Melgar-Quiñonez H, Pérez-Escamilla R (2014). Refinement of the Brazilian Household Food Insecurity Measurement
Scale recommendation for a 14-item EBIA. Rev Nutr.

[18] Associação Brasileira de Empresas de Pesquisas Critérios de Classificação Econômica Brasil..

[19] Young SL, Boateng GO, Jamaluddine Z, Miller JD, Frongillo EA, Neilands TB (2019). The Household Water InSecurity Experiences (HWISE) scale
development and validation of a household water insecurity measure for
low-income and middle-income countries. BMJ Glob Health.

[20] Salles-Costa R, Ferreira AA, Mattos RA, Reichenheim ME, Pérez-Escamilla R, Bem-Lignani J (2022). National trends and disparities in severe food insecurity in
Brazil between 2004 and 2018. Curr Dev Nutr.

[21] Salles-Costa R, Segall-Corrêa AM, Alexandre-Weiss VP, Pasquim EM, Paula NM, Lignani JB (2023). Rise and fall of household food security in Brazil, 2004 to
2022.. Cad Saúde Pública.

[22] Souza FNJ, Bernardes MS, Vieira VCR, Francisco PMSB, Marín-León L, Camargo DFM (2021). (In)segurança alimentar no Brasil no pré e pós pandemia da
COVID-19: reflexões e perspectivas.. InterAmerican Journal of Medicine and Health.

[23] Recine E, Fagundes A, Silva BL, Garcia GS, Ribeiro RCL, Gabriel CG (2020). Reflections on the extinction of the National Council for Food
and Nutrition Security and the confrontation of COVID-19 in
Brazil. Rev Nutr.

[24] Da Mata MM, Neves JA, Medeiros MAT (2022). Hunger and its associated factors in the western Brazilian Amazon
a population-based study. J Health Popul Nutr.

[25] Trivellato PT, Morais DC, Lopes SO, Miguel ES, Franceschini SCC, Priore SE (2019). Food and nutritional insecurity in families in the Brazilian
rural environment: a systematic review.. Ciênc Saúde Colet.

[26] Lignani JB, Palmeira PA, Antunes MML, Salles-Costa R (2020). Relationship between social indicators and food insecurity a
systematic review. Rev Bras Epidemiol.

[27] Sulaiman N, Yeatman H, Russell J, Law LS (2021). A food insecurity systematic review experience from
Malaysia. Nutrients.

[28] McKay FH, Sims A, van der Pligt P (2023). Measuring food insecurity in India a systematic review of the
current evidence. Curr Nutr Rep.

[29] Gebremichael B, Beletew B, Bimerew M, Haile D, Biadgilign S, Baye K (2022). Magnitude of urban household food insecurity in East Africa a
systematic review and meta-analysis. Public Health Nutr.

[30] Garnelo L, Sousa ABL, Silva CO (2017). Regionalização em saúde no Amazonas avanços e
desafios. Ciênc Saúde Colet.

[31] Loschi M (2017). Rural amazônico as peculiaridades das áreas rurais de
florestas. Retratos.

[32] Stoler J, Pearson A, Staddon C, Wutich A, Mack E, Brewis A (2020). Cash water expenditures are associated with household water
insecurity, food insecurity, and perceived stress in study sites across 20
low- and middle-income countries. Sci Total Environ.

[33] Araujo LF, Camargo FP, Torres A, Vernin NS, Andrade RC (2022). Análise da cobertura de abastecimento e da qualidade da água
distribuída em diferentes regiões do Brasil no ano de 2019. Ciênc Saúde Colet.

[34] Santana ABC, Forster AL, Mendes AP, Yamaguchi KL (2021). Análise de dados do Sistema de Informação de Vigilância da
Qualidade da Água para Consumo Humano (Sisagua) no estado do Amazonas,
2016-2020. Vigil Sanit Debate.

[35] Miller JD, Workman CL, Panchang SV, Sneegas G, Adams EA, Young SL (2021). Water security and nutrition current knowledge and research
opportunities. Adv Nutr.

[36] Brewis A, Workman C, Wutich A, Wendy Jepson W, Young S (2019). Household water insecurity is strongly associated with food
insecurity evidence from 27 sites in low- and middle-income
countries. Am J Hum Biol.

[37] Guimarães RM, Andrade FCD (2021). Simpson's paradox a demographic case study of population
dynamics, poverty, and inequality. Ciênc Saúde Colet.

[38] Menezes JA, Confalonieri U, Madureira AP, Duval IB, Santos RB, Margonari C (2018). Mapping human vulnerability to climate change in the Brazilian
Amazon the construction of a municipal vulnerability index. PLoS One.

[39] Alpino TMA, Santos CRB, Barros DC, Freitas CM (2020). COVID-19 and food and nutritional (in)security action by the
Brazilian Federal Government during the pandemic, with budget cuts and
institutional dismantlement. Cad Saúde Pública.

[40] Neves JA, Vasconcelos FAG, Machado ML, Recine E, Garcia GS, Medeiros MAT (2022). The Brazilian cash transfer program (Bolsa Família) a tool for
reducing inequalities and achieving social rights in Brazil. Glob Public Health.

[41] Palmeira PA, Bem-Lignani J, Salles-Costa R (2022). Acesso aos benefícios e programas governamentais e insegurança
alimentar nas áreas rurais e urbanas do Nordeste brasileiro. Ciênc Saúde Colet.

[42] Ministério da Saúde e-Gestor..

